# Decrease of circARID1A retards glioblastoma invasion by modulating miR-370-3p/ TGFBR2 pathway

**DOI:** 10.7150/ijbs.66673

**Published:** 2022-08-08

**Authors:** Baisheng Li, Jiansheng Chen, Yi Wu, Honghai Luo, Yiquan Ke

**Affiliations:** 1 The National Key Clinical Specialty, The Engineering Technology Research Center of Education Ministry of China, Guangdong Provincial Key Laboratory on Brain Function Repair and Regeneration, Department of Neurosurgery, Zhujiang Hospital, Southern Medical University, Guangzhou 510282, China.; 2 Department of Neurosurgery, The Sixth Affiliated Hospital, South China University of Technology, Foshan 528200, China.; 3 Department of Neurosurgery, Huizhou Municipal Central Hospital, Huizhou Shi, China.

**Keywords:** circARID1A, miR-370-3p, TGFBR2, glioblastoma, exosomes

## Abstract

Increasing evidence suggests that circular RNAs (circRNAs) are involved in regulating tumor biological activity. Glioblastoma (GBM) is one of the most lethal diseases characterized by highly aggressive proliferative and invasive behaviors. We aimed to explore how circRNAs influenced GBM biological activity. By circRNA array analysis we found that circARID1A was significantly up-regulated in GBM. Next, we found that circARID1A was up-regulated in GBM tissues and cell lines. Interfering with circARID1A inhibited the migration and invasion of a human GBM cell line U87. By performing dual-luciferase reporter assays, RNA pull-down and fluorescent *in situ* hybridization (FISH), we determined that circARID1A directly bound to miR-370-3p. Moreover, we confirmed that transforming growth factor beta receptor 2 (*TGFBR2*) was the target gene of miR-370-3p by performing RNA pull-down, dual-luciferase reporter assays and western blotting. Further experiments verified that circARID1A promoted GBM cell migration and invasion by modulating miR-370-3p/ TGFBR2 pathway. In addition, we demonstrated that silencing circARID1A restrain the growth of GBM *in vivo*. Finally, we showed that circARID1A was abundant in GBM cell derived exosomes. In conclusion, circARID1A participated in regulating migration and invasion of GBM via modulation of miR-370-3p/ TGFBR2 and thus may be a potential serum biomarker of GBM.

## Introduction

Glioblastoma (GBM) is the most common primary and deadly tumor in the central nervous system, with median survival time of about 14 months 1, even after treatment with surgery, radiotherapy and chemotherapy. Highly invasive behaviors allow GBM cells to infiltrate surrounding brain tissue, which is attributed to incomplete tumor resection and tumor relapse. However, the mechanism underlying GBM's highly aggressive invasive behavior is not clearly known.

Circular RNA (circRNA) is a class of RNA with circular single strand, first described in 1976 2. The biogenesis of circRNAs is not clearly known; long flanking introns 3 and some RNA binding proteins (RBP) such as QKI 4, have been shown to regulate the formation of circRNAs. CircRNAs are classified as exon circRNAs, antisense circRNAs, intron circRNAs, integrated circRNAs 5 and exon-intron circRNAs 6, according to the sequence origin. CircRNAs regulate physiological and pathological process, mainly by acting as a miRNA-sponge 5, 7, binding to RBP 8, 9, binding to RNA Pol II to regulate transcription 6 and via translation into protein 10-12. The expression profile of circRNAs differ between tumor and normal tissues 13, and some circRNAs have been shown to regulate tumor progression 13-15. Importantly, several circRNAs were reported to modulate GBM tumorigenesis and progression; for example, circTTBK2 promotes glioma malignancy by regulating the miR-217/HNF1β/Derlin-1 pathway, while circFBXW7 inhibits GBM tumorigenesis by encoding a protein named FBXW7-185aa. It is likely that additional circRNAs involving GBM progression and tumorigenesis will be discovered.

In this study, we found that circARID1A (hsa_circ_0008494) was up-regulated in GBM tissues and GBM cell lines. Knockdown of circARID1A significantly decreased migration and invasion of tumor cells. By sponging miR-370-3p, circARID1A promoted the expression of transforming growth factor beta receptor 2 (TGFBR2) to facilitate GBM migration and invasion. Furthermore, circARID1A is abundant in GBM cell derived exosomes. These results suggested a novel mechanism underlying the aggressive phenotype of GBM.

## Materials and methods

### Clinical specimens and cell culture

A total of 16 GBM specimens and 9 normal brain tissues (NBT) were collected at Zhujiang Hospital, Southern Medical University, Guangzhou, China, with patients providing informed consent. All methods were performed in accordance with guideline approved by the Ethics Committee of Zhujiang Hospital. The human GBM cell lines U87, U118, LN18 and A172 were purchased from the American Type Tissue Culture Collection, USA. Human astrocyte HEB cells were purchased from GuangZhou Jennio Biotech Co., Ltd. These cells were cultured in DMEM (Gibico, USA) containing 10% FBS, 5% CO_2_, 37°C.

### Microarrays

Total RNA from five GBM tissues and four NBT collected from Zhujiang Hospital was extracted and used to construct circRNA microarrays (Arraystar, USA). CircRNAs with fold changes > 2 and a *p* value < 0.05 were considered differentially expressed. The predictions of potential circRNAs binding miRNAs were made by TargetScan (targetscan.org/vert_71/) and miRnada (microrna.org/microrna/ home.do). Data from the circRNA microarrays is presented in Supplementary materials.

### Transfections

Silencing (si) RNAs and miR-370-3p inhibitor were synthesized by GenePharma (Jiangsu, China). Transfections were performed using Lipofectamine 3000 (Invitrogen, USA). SiRNAs/ miR-370-3p inhibitor and Lipo3000 were mixed in OptiSEM (Gibico, USA) to form a complex. Culture medium was changed 6h after the complex was added to the medium of U87 cells. After 48h, U87 cells were harvested for RNA and protein extraction. The construction of stable U87 cell lines transfected with short hairpin (sh)-circARID1A (containing a puromycin resistance vector) and miR-370-3p inhibitor (containing a hygromycin resistance vector) utilized lentiviral constructs (GenePharma, China). Stably transfected U87 cell lines were selected after puromycin and hygromycin exposure for 2 weeks. Detailed sequences of siRNA and miR-370-3p inhibitor are shown in Supplementary materials.

### Quantitative real-time PCR

Total RNA from tissues and cells was extracted by Trizol reagent (Invitrogen, USA). A total of 1μg RNA was reverse transcribed into cDNA using PrimeScript™ RT Reagent Kit with gDNA Eraser (Takara Bio Inc., Japan). The treatment with RNase R was described in our previous study 16. In brief, 1μg RNA was incubated with 1 U RNase R at 37°C for 10 min. The RNase R (+) and RNase R (-) RNA were subjected to reverse transcription into cDNA. Real-time polymerase chain reaction (RT-PCR) was performed using a SYBR kit (Takara) and the Applied Biosystems 7500 Real-time PCR System. The gene/miR-370-3p expression data were normalized to GADPH/ U6 RNA expression. The primer sequences used in this study are shown in Supplementary Materials.

### Transwell assays

Transwell chambers (Corning FluoroBlok) with an 8μm pore size were used to measure cells migration and invasion. After transfections for 48h, 10^4^ cells suspended in 100μL serum-free medium were seeded into the upper chamber with or without Matrigel (Corning). DMEM containing 10% FBS was added to the lower chamber. After incubation for 48 h in 5% CO_2_, 37°C, cells that moved to the membrane were fixed with 4% paraformaldehyde and stained with DAPI. The number of migrating and invading cells was counting five random fields under light microscopy.

### RNA pull-down

The procedure for RNA-pull down was described in previous study 17. In brief, 100 pmol biotin-labeled circARID1A probe or biotin-labeled miR-370-3p were incubated with 50μL streptavidin beads at 4°C overnight. A total of 2×10^7^ U87 cells were lysed and then incubated with the bead-biotin complex at room temperature (RT) for 1 h. After wash with wash buffer, the product was subjected to RNA extraction and RT-PCR.

### Dual-luciferase reporter assay

The sequence of circARID1A (WT1) and corresponding miR-370-3p mutants (Mut1), as well as the sequence for the 3'-UTR of TGFBR2 (WT2) and corresponding miR-370-3p binding site mutant (Mut2) were cloned into the psiCHECK-2 plasmid (GenePharma). The 293T cells were cultured and co-transfected with miR-370-3p mimic and WT1/Mut1 or WT2/Mut2 plasmid. After 48 h, luciferase activity was measured with Firefly Renilla Luciferase Kit (Promega, USA). Sequences of plasmids are shown in Supplementary materials.

### Fluorescence in situ hybridization

A digoxin- labeled circARID1A probe and a biotin- labeled miR-370-3p probe were synthesized by Guangzhou Exon Biotechnology Co.,ltd. For FISH in cells, 2×10^4^ cells were seeded on a slide overnight and fixed with 4% paraformaldehyde at RT for 20 min. Then the slide was incubated with 3% H_2_O_2_ at RT for 10 min and washed with phosphate-buffered saline (PBS). Target retrieval was performed with citrate buffer (pH=6.0) at 95°C for 30 min. After washing with PBS, the slide was incubated with 0.01% pepsin at 37°C for 15 min. Next, the slide was incubated with Hybridization Buffer (Exon Biotechnology, China) at 37°C for 30 min, then incubated with probe in Hybridization Buffer (1:50) at 37°C overnight. Then the slide was washed with 2 × saline-sodium citrate buffer (SSC) at 42°C. After blocking with 3% bovine serum albumin (BSA), the slide was incubated with anti-digoxin horseradish peroxidase (HRP)/anti- biotin HRP at 37°C for 1 h. The slide was stained with tyramide signal amplification kit (Exon Biotechnology, China) at RT for 15 min. Finally, the slide was incubated with DAPI at RT for 20min. Images were captured by fluorescence microscope (Nikon 80i, Japan). .

In order to measure the level of circARID1A in GBM, we purchased a tissue microarray (N095Ct01, Bioaitech Co., Ltd, China) containing 85 GBM tissues and 10 NBT. The slide was deparaffinized with xylene twice, then re-hydrated with decreasing concentrations of alcohol (100%, 85%, 75%) and washed with PBS. The method for detecting circARID1A signal was the same as described above. The fluorescence signals were scanned using a Pannoramic DESK (P250, 3D HISTECH, Hungary). The intensity of fluorescence was measured as the mean value of the integrated optical density (IOD; mean IOD=IOD (sum)/area (sum). The IOD value of each specimen was obtained using Image J.

### Western blotting

Cells were lysed in RIPA (Solarbio, China) and the lysates were denatured with loading buffer at 100°C for 10 min. Then the samples were subjected to SDS-PAGE as previous described 18. The primary antibodies using in our study were raised against TGFBR2 (Bioss, China), E-Cadherin, N-Cadherin, MMP2, MMP-9, MMP-14 and GAPDH (Proteintech, China).

### Intracranial nude mouse glioma xenograft model

The intracranial nude mouse glioma xenograft model was established as previous described 18, 19. In brief, 2×10^5^ U87-luc cells stably transfected with sh-negative control (NC) or sh-circARID1A were injected stereotaxically into the right hemicerebrum of 4- to 6- week-old female nude mice (n = 5; BALB/c-nu, Guangdong Medical Laboratory Animal Center, China). On day 3, day 10 and day 15, tumor growth was monitored by *in vivo* imaging system (Lumina, USA) after an intraperitoneal injection of luciferase substrate-D-luciferin (YEASEN, China). On day 15, tumors were harvested and subjected to immunohistochemistry assay.

### Immunohistochemistry

The tumors harvested from the mice were fixed with 4% paraformaldehyde and embedded in paraffin. The sections were cut 4- μm thick and mounted on slides. The slides were deparaffinized, rehydrated and antigen retrieval as described above. After blocking with 3% BSA, anti-TGFBR2 (Bioss) was incubated at 4°C overnight. Subsequent, the secondary antibody conjugated HRP (BOSTER, China) was incubated with the sections at RT for 1 h. 3,3-diaminobenzidine (DAB) solution was used to detect staining, with hematoxylin as the counterstain. Images of five randomly chosen fields from each group were captured by microscope (Nikon 80i, Japan) and the mean IOD value using Image J.

### Isolation and characterization of exosomes

Exosomes were isolated from cell culture medium using the method of ultracentrifugation. In brief, cell debris was removed by centrifugation at 2000 × *g* for 20 min and the supernatant was collected. Then the supernatant was subjected to centrifugation at 10,000 × *g* for 30 min and 100,000 × *g* for 90 min. The supernatant was discarded, and exosomes were washed with PBS at 100,000 × *g* for 60 min. The exosomes were resuspended in 100μL PBS. Images of the isolated exosomes were captured by transmission electron microscopy (TEM; Hitachi HT7650, Japan) as previously described 20. In brief, the isolated exosomes were fixed with 2% paraformaldehyde and then 5 -10 μL isolated exosomes were added onto a copper grid by filter paper. When the copper grid was completely dry, the sample was stained with 2% uranyl acetate, and the sample was measure at 100 Kev. The size distribution of exosomes was assessed using nanoparticle tracking analysis (NTA) by Nano-Zs instrument (Malvern Panalytical, UK) according to the manufacturer's instructions.

### Statistical analysis

Statistical analysis was performed using SPSS 20.0 (IBM). Statistical significance between groups was determined by *t-test* Mann-Whitney U test or analysis of variance (ANOVA) as appropriate. *P* < 0.05 was considered statistically significant. Results are shown as mean ± SEM.

## Results

### Levels of circARID1A were elevated in GBM tissues and GBM cell lines

**Figure 1:**
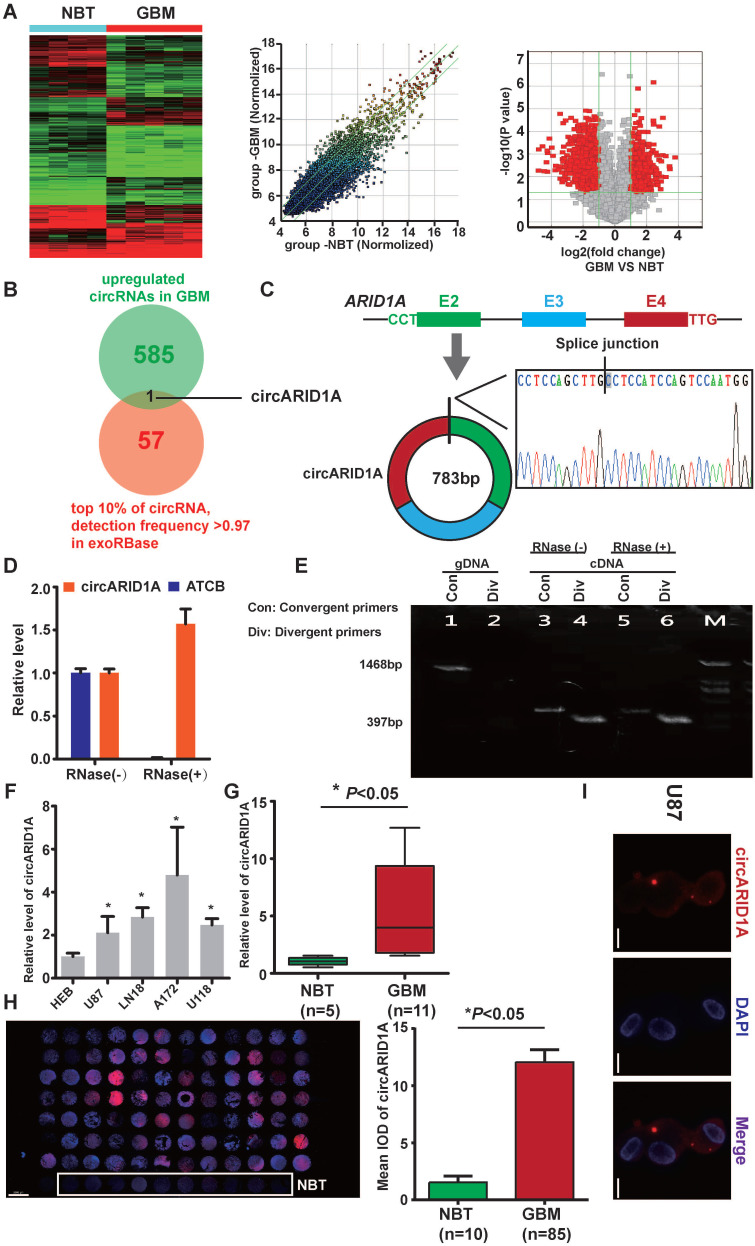
** The circARID1A was upregulated in GBM tissues and GBM cell lines**. **A**. Heat map (left), scatter plot (middle) and volcano plot (right) of circRNAs in five GBM tissues vs. four NBT. **B**. Venn diagram for circRNAs upregulated in GBM tissues showing the top 10% ranked by expression with detection frequencies greater than 0.97 in exoRBase. **C**. Upper: Schematic illustration of circARID1A formation. Lower: Sanger sequence demonstrated the correct splice junction. **D**. circARID1A was resistant to RNase R treatment, whereas linear RNA ATCB was degraded. (n = 3, mean ± SEM). **E**. Images of agarose gels showing amplified of cDNA by divergent primers and resistance to RNase R treatment.** F**. The expression of circARID1A in GBM cell lines and astrocyte-HEB cells. Compared with HEB cells, circARID1A was significantly upregulated in GBM cell lines. n =3, *p < 0.05, ANOVA.**G**. circARID1A was significantly upregulated in GBM compared with NBT in our cohort. (11 GBM vs. 5 NBT, *p < 0.05, t test). **H**. circARID1A was significantly upregulated in GBM compared with NBT by performing FISH in tissue microarray, scale bar, 2000μm, (85 GBM vs. 10 NBT, *p < 0.05, *t*-test). **I**. FISH images showed that circARID1A was in both the cytoplasm and nucleus, but was primarily localized in the cytoplasm of U87 cells, scale bar, 100μm.

From circRNA arrays of five GBM tissues and four NBT, expression of circRNAs expression was different. Among the differentially expressed circRNAs, 585 were significantly up-regulated and 985 were significantly down-regulated in GBM tissues, compared with NBT (fold change >2, *p*<0.05); Figure 1A). By screening in exoRBase 21 (a dataset characterizes the abundance of mRNA, lncRNA and circRNA in human blood exosomes), there were 57 circRNAs ranked in the top 10% expression with detection frequency greater than 0.97. Combined with our data from circRNA arrays, we found that circARID1A (hsa_circ_0008494) was highly abundant and could be detected in human blood exosomes (Figure 1B). Therefore, we decided to focus on this circRNA. CircARID1A originates from exons 2 to 4 of gene *ARID1A*, and its mature length after splicing is 783bp (Figure 1C). As expected, circARID1A was resistant to RNase R treatment (Figure 1D). By performing agarose gel electrophoresis after PCR, circARID1A was amplified from cDNA by divergent primers and was resistant to RNase R treatment (Figure 1E). Next, we verified that circARID1A was up-regulated in GBM tissues and GBM cell lines, compared with NBT and HEB cells (Figure 1F-G). Moreover, we measured the expression of circARID1A by performing FISH in tissue microarrays, and the result showed that circARID1A was significantly up-regulated in GBM tissues, compared with NBT (Figure 1H). In addition, by performing FISH in U87 cells, we showed that circARID1A mainly localized to cytoplasm (Figure 1I). These data suggested that circARID1A may promote GBM progression and serve as a potential serum biomarker of GBM.

### Decrease circARID1A inhibited GBM migration and invasion *in vitro*

**Figure 2:**
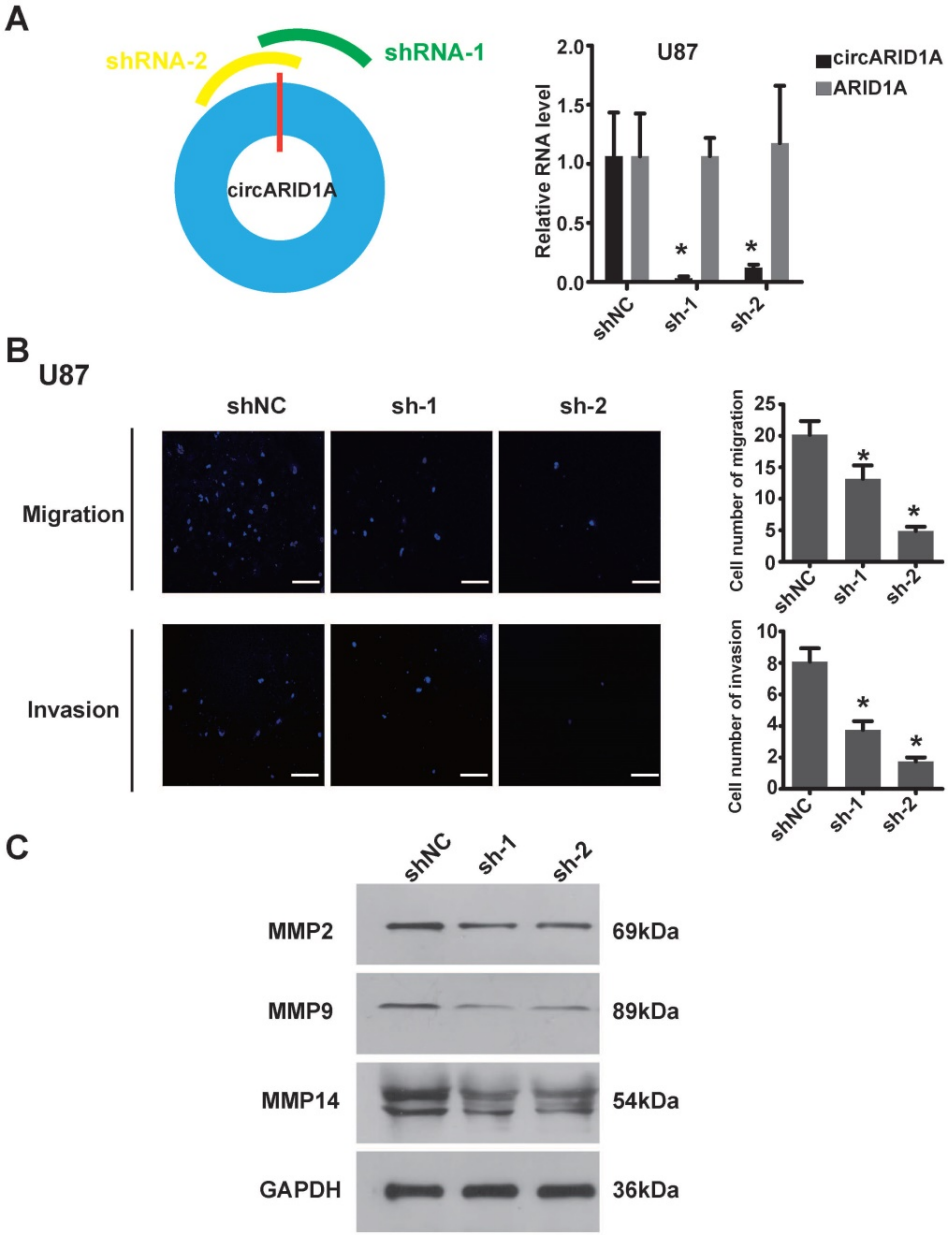
**Decrease in circARID1A inhibited U87 cell migration and invasion *in vitro***. **A**. Left: Schematic illustration of two siRNAs specially targeting the junction of circARID1A; Right: Stable interference system for circARID1A by lentiviral transfection in U87 cells. n = 3,*p < 0.05, *t*-test. **B**. Left: Images of Transwell assays of U87 cells, scale bar, 100μm; Right: Analyses of Transwell assay results indicated that decrease in circARID1A significantly inhibited U87 cell migration and invasion. n = 5,*p < 0.05, ANOVA. **C.** Images of western blotting showed that the expressions of MMPs decreased after silencing circARID1A.

In order to explore the function of circARID1A in GBM, we designed two shRNAs specially targeting the junction of circARID1A without interference from ARID1A mRNA. The efficiency of two shRNAs was validated by performing RT-PCR (Figure 2A). Next, by performing transwell assays, knockdown of circARID1A was found to significantly inhibit U87 cells (Figure 2B) and U118 cells (Supplementary Figure 1A) migration and invasion. Furthermore, the expression of MMPs decreased when circARID1A was silenced (Figure 2C, Supplementary Figure 1B). These results suggested that circARID1A promoted invasion in GBM.

### CircARID1A directly bound to miR-370-3p

**Figure 3:**
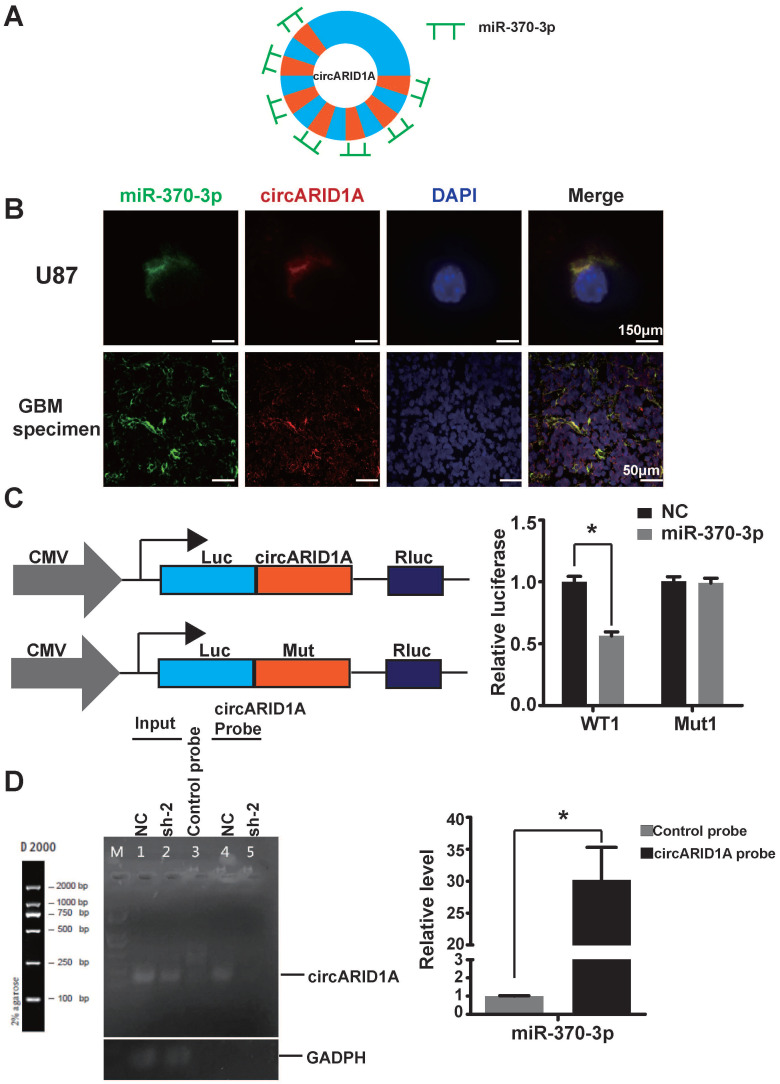
** The circARID1A directly bound to miR-370-3p**. **A**. Schematic illustration of the seven binding sites for miR-370-3p in circARID1A. **B**. Images of FISH showed that circARID1A and miR-370-3p co-localized in the cytoplasm of U87 cells and GBM specimen, scale bar, 150μm (upper) 50μm (lower). **C**. Dual-luciferase reporter assay showed that co-transfection of WT1 and mimic miR-370-3p markedly decreased luciferase activity in 293T cells, whereas luciferase activity did not change in 293T cells when miR-370-3p binding sites in circARID1A were mutated. n = 3, *p < 0.05, *t*-test. **D**. Left: Images of agarose gels showed that biotin-labeled probe specifically targeted circARID1A, with oligo probe as a negative control; Right: Histogram of qPCR of RNA pull-down assay. Compared with oligo probe, miR-370-3p in the group probe was significantly enriched. n = 3, *p < 0.05, *t*-test.

Previous studies have demonstrated that circRNA could act by sponging miRNAs 13, 22, 23, binding to RBP 9, 24 and encoding peptides 12. Given that circARID1A localized to the cytoplasm and lacked an internal ribosome entry sequence (IRES), which is essential to encode peptides, we hypothesized that circARID1A may function as a miRNA sponge. The result of bioinformatics analysis (TargetScan and miRnada) showed that circARID1A contains seven binding sites for miR-370-3p (Figure 3A). These data indicated that circARID1A may act through interaction with miR-370-3p.

By performing FISH, we demonstrated that circARID1A co-localized with miR-370-3p in the cytoplasm of U87 cells and GBM specimens (Figure 3B). Next, we investigated whether circARID1A could bind to miR-370-3p. We constructed a wild type (WT1) dual-luciferase reporter plasmid by inserting the sequence of circARID1A, and a mutant plasmid (Mut1) which seven binding sites of miR-370-3p for circARID1A were mutated. After co-transfection with miR-370-3p mimic, the activity of luciferase in the WT1 group was significantly decreased, whereas this regulation vanished in the Mut1 group (Figure 3C). These results suggested that circARID1A bound to miR-370-3p.

Next, we aimed to confirm whether circARID1A could directly bind to miR-370-3p. A biotin-labeled circARID1A probe was designed to pull down circARID1A in U87 cells. The pull-down efficiency of circARID1A was significantly enhanced using the circARID1A probe, compared with a control oligo probe. The products of pull-down assay were subjected to RT-PCR and the data showed that miR-370-3p was abundantly pulled-down by circARID1A (Figure 3D). These results demonstrated that circARID1A could directly bind to miR-370-3p.

### The circARID1A promoted migration and invasion by modulating miR-370-3p/TGFBR2 *in vitro*

**Figure 4:**
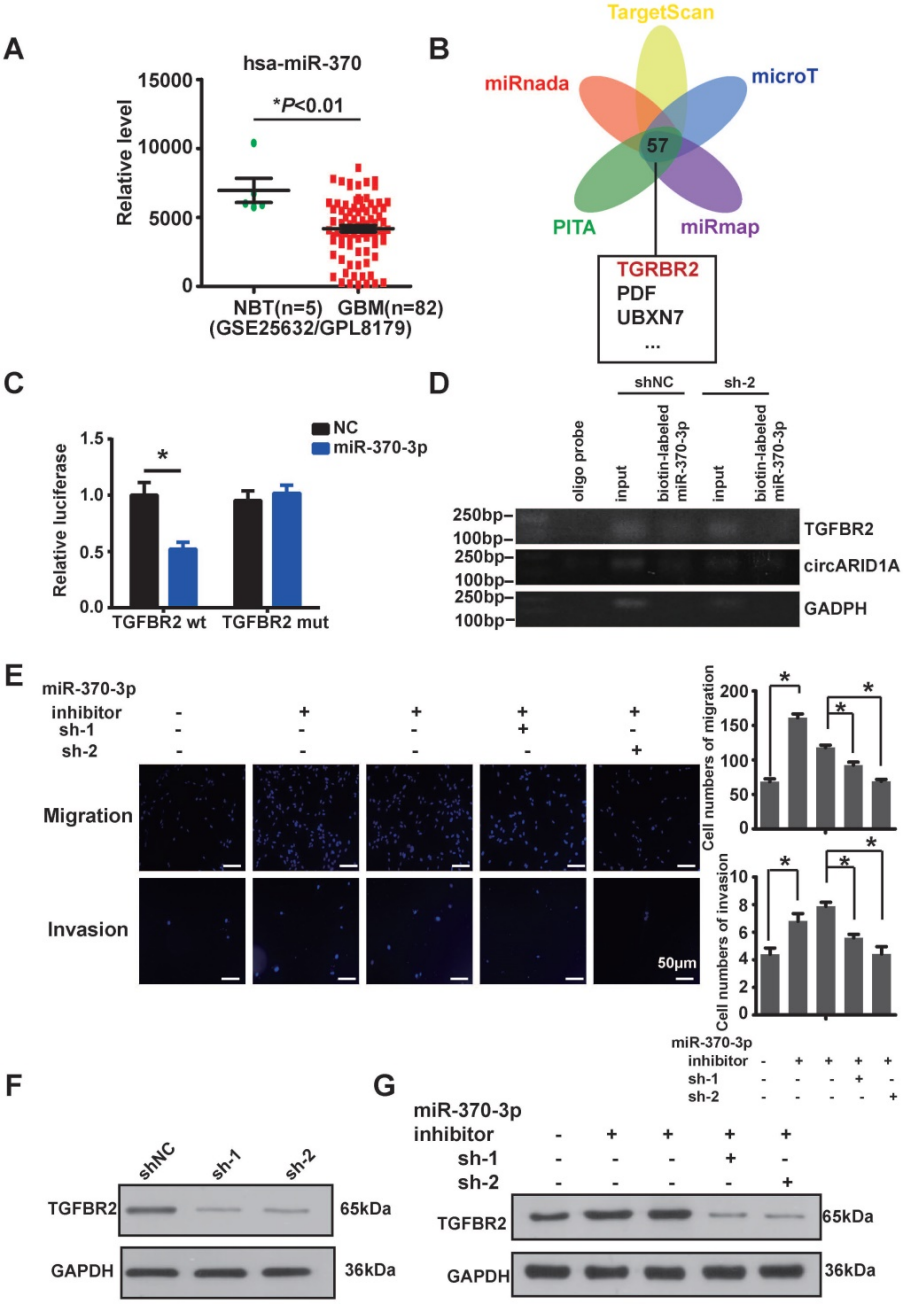
** The circARID1A promoted migration and invasion via modulation the miR-370-3p/TGFBR2 pathway* in vitro***. **A**. Data from GEO datasets (GSE25632/ GPL8179) indicated that hsa-miR-370 was significantly downregulated in GBM compared with NBT. *p < 0.05, *t*-test. **B**. Venn diagram for predicted targets of miR-370-3p by ENCORI. **C**. Dual-luciferase reporter assay showed that co-transfection of WT2 and mimic miR-370-3p markedly decreased luciferase activity in 293T cells, whereas luciferase activity did not change in 293T cells when miR-370-3p binding sites in circARID1A were mutated. n = 3, *p < 0.05, *t-*test). **D**. Images of agarose gel showed that biotin-labeled miR-370-3p pulled down circARID1A and TGFBR2, with oligo probe as a negative control. TGFBR2 and circARID1A were markedly abundant in group biotin-labeled miR-370-30. E. Left: Images of Transwell assays of U87 cells; Right: Analyses of Transwell assays results indicated that transfections of miR-370-3p inhibitor promoted U87 cell migration and invasion, while silencing circARID1A suppressed the promotion. scale bar, 50μm n = 5,*p < 0.05, ANOVA. **F**. Images of western blotting showed that expression of TGFBR2 protein was decreased after silencing circARID1A. G. Images of western blotting showed that transfections of miR-370-3p inhibitor promoted TGFBR2 expression, while silencing circARID1A suppressed the promotion.

In order to explore the regulation of miR-370-3p in GBM, we analyzed data from the GEO dataset, which showed that miR-370 was significantly down-regulated in GBM, compared with NBT (Figure 4A), which suggested that miR-370-3p could act as a tumor suppressor. MiRNAs are 19-23nt-long noncoding RNAs that regulate various biological processes including tumor progression by targeting 3'-UTR of mRNA 25. By searching in ENCORI dataset 26, we found that 57 genes were mutually predicted by TargetScan, PITA, miRanda, microT and miRmap (Figure 4B). Among these genes, we focused on TGFBR2, which is one of the receptors of TGF-β1 and associated with tumor migration and invasion 27, 28. We hypothesized that circARID1A promoted GBM invasion by modulating the miR-370-3p/ TGFBR2 pathway.

In order to confirm whether miR-370-3p bound to TGFBR2, we constructed a wild type (WT2) dual-luciferase reporter plasmid by inserting the 3'-UTR sequence of TGFBR2, and also constructed a mutant plasmid (Mut2) which the binding site for miR-370-3p in TGFBR2 3'-UTR was mutated. After co-transfections with miR-370-3p mimic, the luciferase activity in group WT2 was markedly decreased whereas the luciferase activity did not change in group Mut2 (Figure 4C). This result suggested that miR-370-3p bound to the 3'-UTR of TGFBR2. In order to verify whether miR-370-3p could directly target TGFBR2, we performed pull-down assays with the biotin-labeled miR-370-3p in U87 cells. The data showed that TGFBR2 and circARID1A were pulled-down in abundance by miR-370-3p (Figure 4D). Next, by performing transwell assays, we demonstrated that migration and invasion of U87 cells (Figure 4E) and U118 cells (Supplementary Figure 1C) were increased after transfection with miR-370-3p inhibitor. However, this regulation vanished after co-transfections of sh-circARID1A. These results showed that circARID1A promoted GBM cell migration and invasion by sponging miR-370-3p. Moreover, the expression of TGFBR2 protein was decreased after silencing circARID1A (Figure 4F, Supplementary Figure 1D). Importantly, the expression of TGFBR2 protein was markedly increased after co-transfection with miR-370-3p inhibitor, while this regulation was reversed by co-transfection of sh-circARID1A (Figure 4G). These results indicated that circARID1A promoted GBM cell migration and invasion via modulation of the miR-370-3p/TGFBR2 pathway *in vitro*.

### Silencing of circARID1A inhibited tumor growth *in vivo*

**Figure 5:**
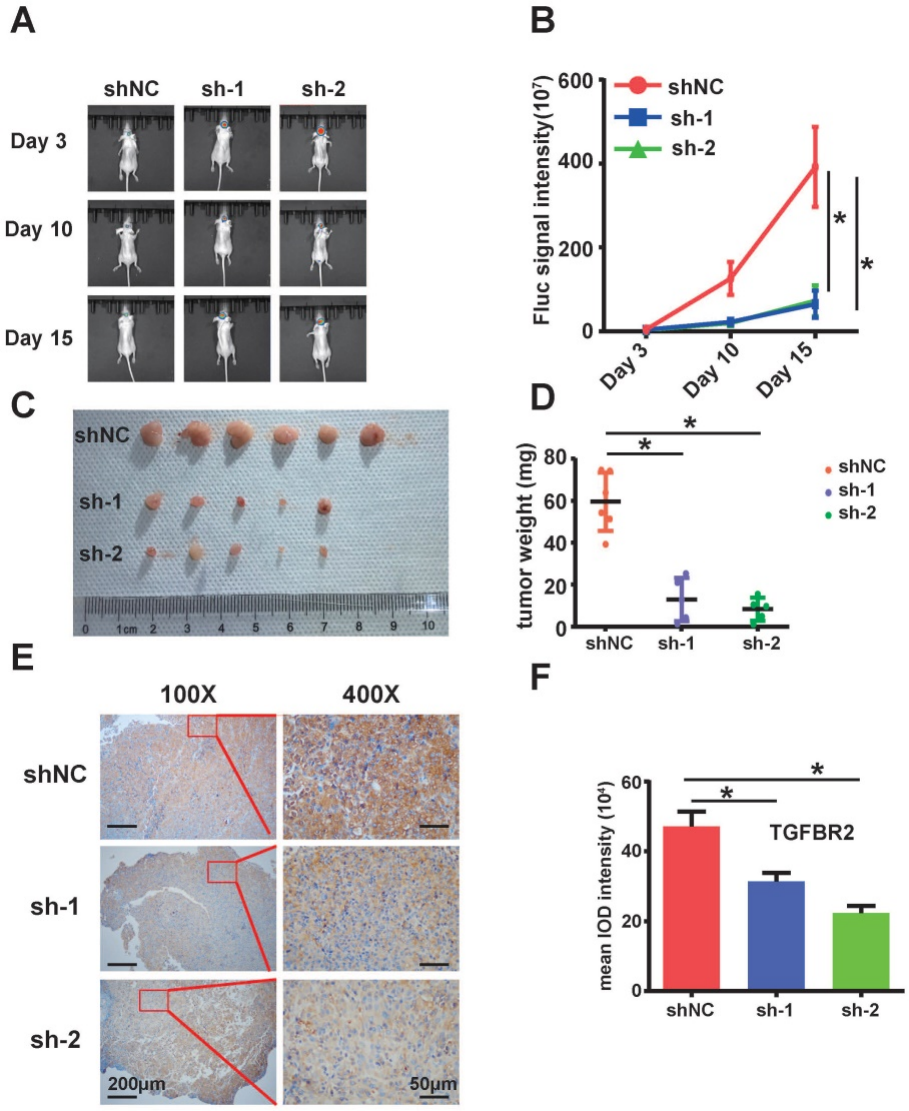
** Silencing of circARID1A significantly retarded GBM growth *in vivo***. **A**. Images of intracranial glioma xenograft in BALB/c-nu after injection of D-luciferin using *in-vivo* imaging system. **B**. Results showing that the growth rate was significantly decreased in group sh-circARIDIA compared with group shNC. n =5 *p < 0.05, ANOVA. **C**. Images of tumor harvested in day 15. **D**. Tumor weights in group sh-circARID1A were significantly decreased compared to weight in group shNC. n = 5, *p < 0.05, *t-*test. **E, F**. Immunohistochemistry Images showing that the expression of TGFBR2 markedly decreased when circARID1A was silenced, n = 5, *p < 0.05, ANOVA

Next, we examined the effect of circARID1A on GBM *in vivo*. We utilized stably transfected U87-Luc-shNC and U87-Luc-sh-circARID1A cells to establish a nude mouse intracranial glioma xenograft model as described in our previous study 19. The result suggested that the tumor growth rate in the sh-circARID1A group was slower than in the shNC group (Figure 5A-B). Moreover, tumor weights in the sh-circARID1A group were significantly decreased compared with in the shNC group (Figure 5C-D). In addition, by performing immunohistochemistry assays, we showed that the expression of TGFBR2 in the sh-circARID1A group was markedly reduced compared to in the shNC group (Figure 5E-F). These results suggested that silencing circARID1A inhibited tumor growth by decreasing TGFBR2 expression *in vivo*.

### CircARID1A was abundant in GBM cell- derived exosomes

**Figure 6:**
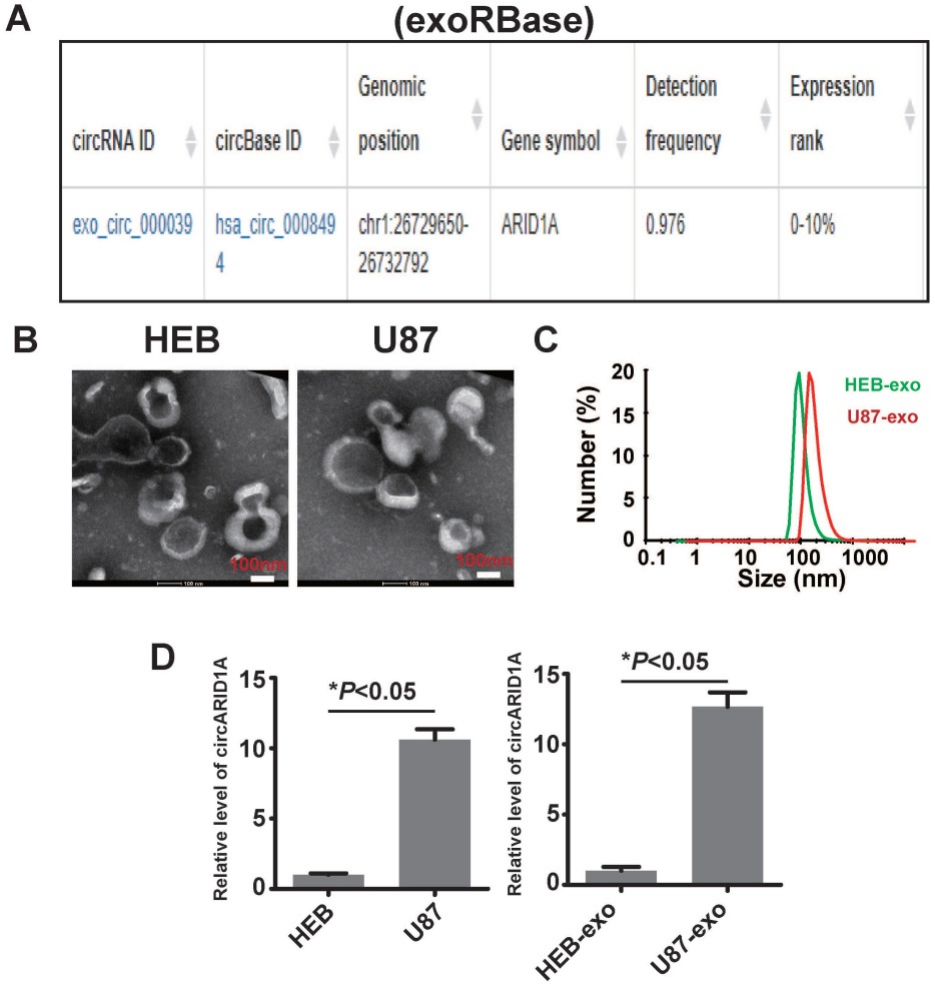
** The circARID1A was abundant in U87 cell- derived exosomes**. **A**. Dataset of exoRBase suggested that the abundance of circARID1A was in the top 10% of all circRNAs in human serum exosome. **B**. Micrographs of isolated exosomes (left: HEB exosomes; right: U87 exosomes) obtained by TEM. **C**. Size distribution of isolated exosomes by NTA. **D**. The abundance of circARID1A in U87 cells and U87 exosomes was significantly higher than in HEB cells and HEB exosomes, respectively, *P<0.05, *t*-test.

A previous study described how circRNA was enriched in exosomes and the abundance of exo-circRNA was correlated with the abundance of circRNA in tumor tissue 29. By searching exoRBase 21, we found that the abundance of circARID1A was in the top 10% of all circRNA content in human serum exosomes (Figure 6A). Next, we isolated exosomes from FBS-free medium cultured with HEB and U87 cells. The image obtained by TEM showed that the particles isolated from HEB and U87 cells medium contained vesicles isolated from HEB and U87 cell culture medium had a diameter of 30-120nm (Figure 6B). The analysis of size distribution of these exosomes revealed that the average diameter of vesicles derived from HEB and U87 cells was 112.6 nm and 126.2 nm, respectively (Figure 6C). By performing RT-PCR, we demonstrated that the abundance of circARID1A was increased in U87 exosomes, compared to HEB exosomes (Figure 6D). This result suggested that circARID1A may be able to serve as a serum biomarker of GBM.

## Discussion

GBM is a lethal disease, characterized by highly aggressive biological behavior. The mechanism underlying its invasive ability is not clearly known. Recently, many studies have reported that epigenetic aberrations may be responsible for alterations in GBM biological behavior 30-34. Canonical epigenetic modifications include DNA methylation, histone acetylation, chromosome remodeling and non-coding RNA.

The circRNA is a class of RNA with a circular structure, and its biogenesis is associated with long flanking introns 3 or splicing factors 4, 35. The circRNAs are a new type epigenetic modification which participates in regulation via a different mechanism. Since Memczak et al. reported that CDR1as could regulate development of midbrain by sponging miR-7 5, researchers have found many circRNAs can act as miRNA sponges and elevate the expression of downstream targets, such as circHIPK3 13, 36, 37, circBIRC6 38. The circRNAs have also been found to bind to RBP to facilitate the RBP transposition between cytoplasm and nucleus, including circFOXO3 39 and circAmotl1 8, 24. Interestingly, some circRNAs can generate polypeptides driven by IRES 12, 40, 41 or RNA N -m6A modification 42. CircRNAs are also involved in the regulation of transcription. Exon-intron circRNAs can interact with U1 snRNP and RNA Pol II to promote transcription of parent gene 6. In addition, circRNAs function in regulating alternative splicing. CircMBL is generated from the second exon of MBL/MBNL1, which is a splicing factor. MBL can bind to circMBL and its flanking introns to promote circMBL biogenesis, thus, circMBL can regulate pre-mRNA splicing via its interaction with MBL 43. CircURI1 has been proved to interact with hnRNPM to modulate alternative splicing in gastric cancer^44^. The CircRNAs have been proved to influence tumor progression 14, 45, 46, but how circRNAs regulate biological behavior of GBM requires further elaboration.

In this study, by examining circRNA microarrays with five GBM and four NBT tissues, we found that the profile of circRNA expression was different between tissues. Among the circRNAs up-regulated in GBM, circARID1A was abundant in human plasma exosomes as found by searching exoRBase, and therefore was promising as a serum or plasma biomarker of GBM, so we selected it as our research focus. Silencing circARID1A significantly inhibited U87 cell migration and invasion. However, the mechanism underlying circARID1A promotion GBM cell migration and invasion still needed elucidation. By performing FISH, we showed that circARID1A localized to the cytoplasm, and lacked the IRES sequence which is essential for coding protein, so we hypothesized that circARID1A may function by sponging miRNA. Bioinformatic analysis suggested that circARID1A could bind to miR-370-3p. By performing dual-luciferase assays, we verified that circARID1A bound to miR-370-3p, and further confirmed the direct binding of circARID1A to miR-370-3p by RNA pull-down experiments. Because several studies have reported that miR-370-3p acted as a tumor suppressive factor 47, 48, we asked how miR-370-3p influence GBM biological behavior. By analyzing data from the GEO dataset, we confirmed that expression of miR-370-3p was lower in GBM than in NBT. In order to determine the target gene of miR-370-3p, we identified 60 genes mutually predicted by TargetScan, PITA, miRanda, microT and miRmap in the ENCORI dataset. Among these genes, we focused on TGFBR2, which is associated with tumor migration and invasion 27, 28. Further research indicated that transfection with miR-370-3p inhibitor significantly promoted U87 cell migration and invasion by increasing the expression of TGFBR2, while silencing circARID1A suppressed this behavior and expression of TGFBR2. In addition, by establishing an intracranial glioma xenograft model, we found that silencing circARID1A inhibited tumor growth. In summary circARID1A promoted GBM invasion via modulation of the miR-370-3p/TGFBR2 pathway.

CircRNAs are promising in liquid biopsy for tumor diagnosis. A previous study indicated that the circRNA profile of tumor exosomesresembled that of tumor themselves 29. Therefore, we collected exosomes derived from HEB cells and U87 cells. By performing RT-PCR, we showed that circARID1A was significantly enriched in U87 cell exosomes compared to HEB cell exosomes. This result suggested that circARID1A had the potential to serve as a GBM serum biomarker, but further research will be required to verify this hypothesis.

In conclusion, our study demonstrated that circARID1A promoted GBM migration and invasion by modulating the miR-370-3p/ TGFBR2 pathway, and may serve as a potential serum biomarker of GBM.

## Supplementary Material

Supplementary information, figure and table.
