# Supplementary material for: Decrease of circARID1A retards glioblastoma invasion by modulating miR-370-3p/ TGFBR2 pathway
Source: Int J Biol Sci. 2022 Aug 8;18(13):5123–35. doi: 10.7150/ijbs.66673 (PMC9379412; doi:10.7150/ijbs.66673)
Supplement: Supplementary file 1 — Supplementary information, figure and table. [file ijbsv18p5123s1.pdf]

Transfections	
	sequence
Si-circARID1A-1	TTGCCTCCATCCAGTCCAA
Si-circARID1A-2	GCCTCCATCCAGTCCAATG
Hsa-miR-370-3p mimic	GCCUGCUGGGGUGGAACCUGGU<br>ACCAGGUUCCACCCCAGCAGGC
Hsa-miR-370-3p inhibitor	ACCAGGUUCCACCCCAGCAGGC
Primers	
circARID1A-F-con	CCCCTCATGCCCAACCTTCGT
circARID1A-R-con	TGCCCTCCCTTACTGGAGGTCATTG
circARID1A-F-div	AGATTCATTTGGGTCTCAGGC
circARID1A-R-div	ATTGGACTGGATGGAGGCA
ARID1A-F	GGACCTCTATCGCCTCTATGTG
ARID1A-R	GGACTTCTTGGAATCAGCAG

Dual-luciferase reporter plasmid of TGFBR 3'UTR

Predicted binding sites:

	Predicted consequential pairing of target region (top) and miRNA (bottom)	Site type	Context++ score	Context++ score percentile	Weighted context++ score	Conserved branch length	P_{CT}
Position 62-69 of TGFBR2 3' UTR<br>hsa-miR-370-3p	5' ...ACCAAAGAACAGAGGCAGCAGGA... ||||||| 3' UGGUCCAAGGUGGGGUCGUCCG	8mer	-0.03	58	-0.03	4.245	N/A
Position 154-160 of TGFBR2 3' UTR<br>hsa-miR-370-3p	5' ...AAGCAGAAACAACAGCAGCAGGG... ||||||| 3' UGGUCCAAGGUGGGGUCGUCCG	7mer-m8	-0.05	67	-0.05	3.365	N/A

WT:

CTCTTCTGGGGCAGGCTGGGCCATGTCCAAAGAGGCTGCCCCTCTCACCA AAGAACAGAGGCAGCAGGAAGCTGCCCCTGAACTGATGCTTCCTGGAAA ACCAAGGGGGTCACTCCCCTCCCTGTAAGCTGTGGGGATAAGCAGAAACA

ACAGCAGCAGGGAGTGGGTGACATAGAGCATTCTATGCCTTTGACATTGT
CATAGGATAAGCTGTGTTAGCACTTCCTCAGGAAATGAGATTGATTTTTAC
AATAGCCAATAACATTTGCACTTTATTAATGCCTGTATATAAATATGAAT

Mut:

CTCTTCTGGGGCAGGCTGGGCCATGTCCAAAGAGGCTGCCCCTCTCACCA
AAGAACAGAGGagatgcagAGCTGCCCCTGAACTGATGCTTCCTGGAAAACC
AAGGGGGTCACTCCCCTCCCTGTAAGCTGTGGGGATAAGCAGAAACAACA
GtttcagtGAGTGGGTGACATAGAGCATTCTATGCCTTTGACATTGTCATAGGA
TAAGCTGTGTTAGCACTTCCTCAGGAAATGAGATTGATTTTTACAATAGCC
AATAACATTTGCACTTTATTAATGCCTGTATATAAATATGAAT

Applied Biosystems

GeneMapper 4.0

CellLineAuthentication-0326

Sample File	Sample Name	Panel	SQI	OS	SQ
09_A02_CellLineAuthentication-1-0402.FSA	6	STR Profile 1-human			

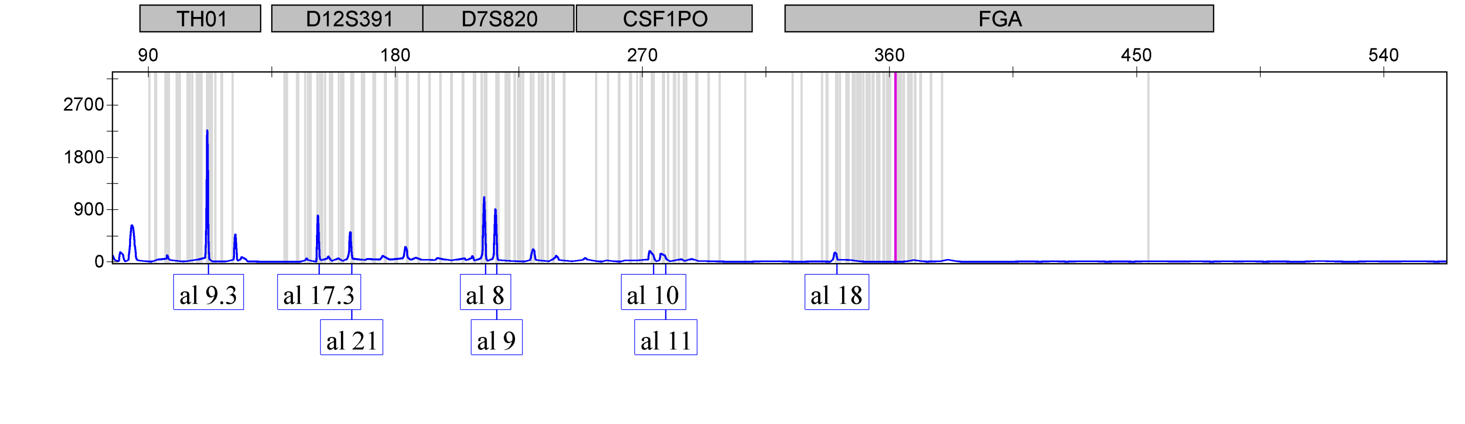

09_A02_CellLineAuthentication-1-0402.FSA	6	STR Profile 1-human			

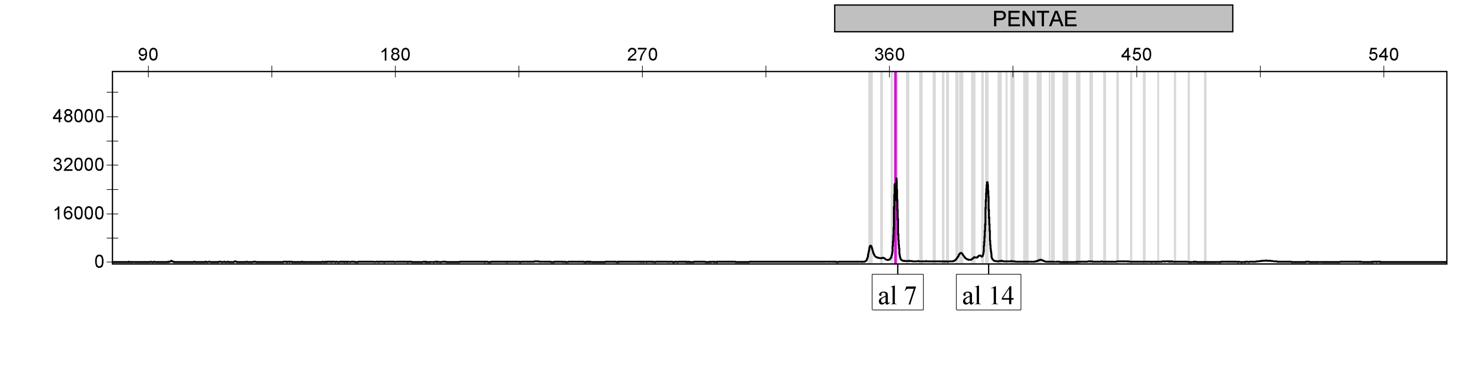

25_A04_CellLineAuthentication-1-0402.FSA	6	STR Profile 2-human			

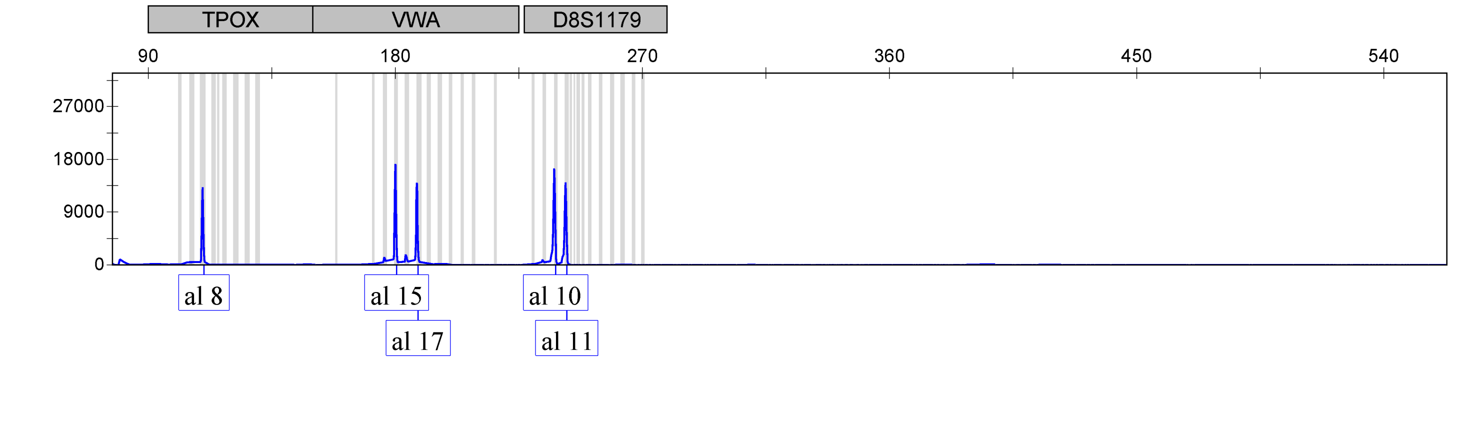

Sample File	Sample Name	Panel	SQI	OS	SQ
25_A04_CellLineAuthentication-1-0402.FSA	6	STR Profile 2-human			

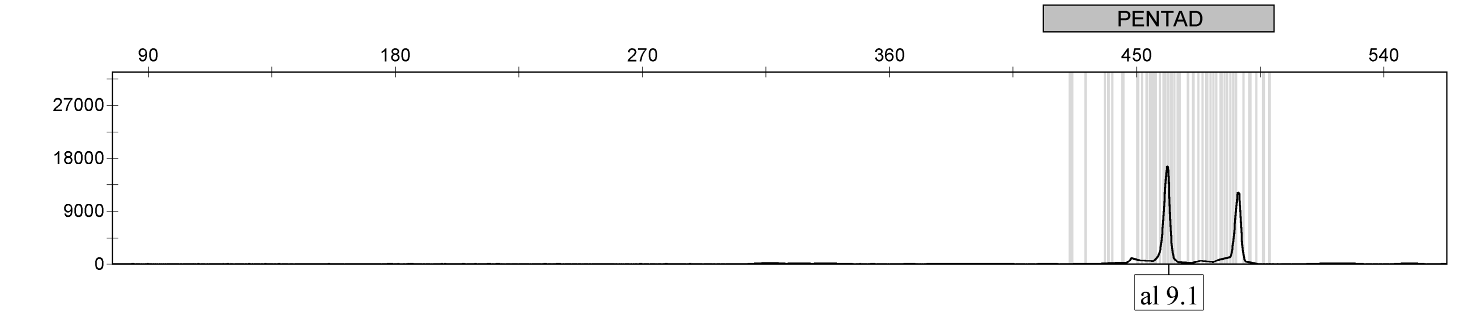

Sample File	Sample Name	Panel	SQI	OS	SQ
41_A06_CellLineAuthentication-1-0402.FSA	6	STR Profile 3-human			

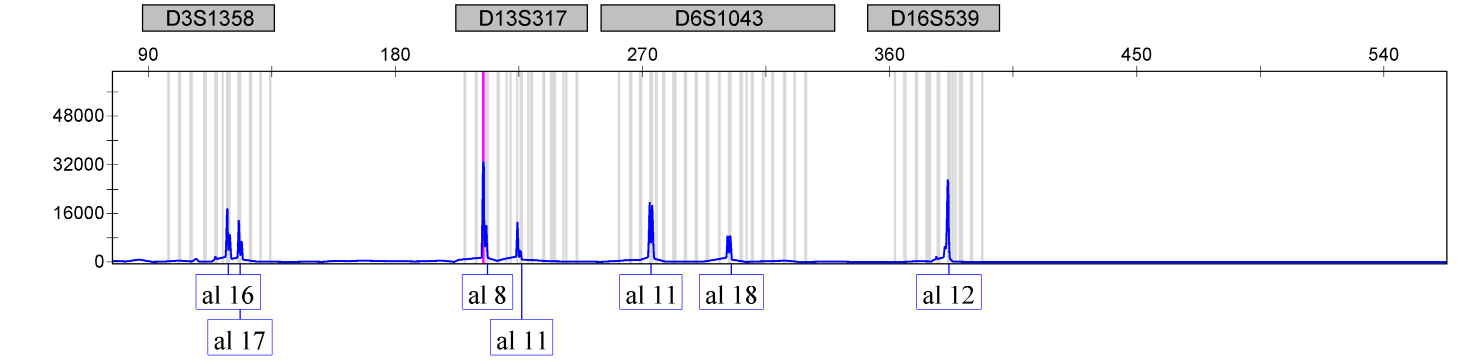

Sample File	Sample Name	Panel	SQI	OS	SQ
41_A06_CellLineAuthentication-1-0402.FSA	6	STR Profile 3-human			

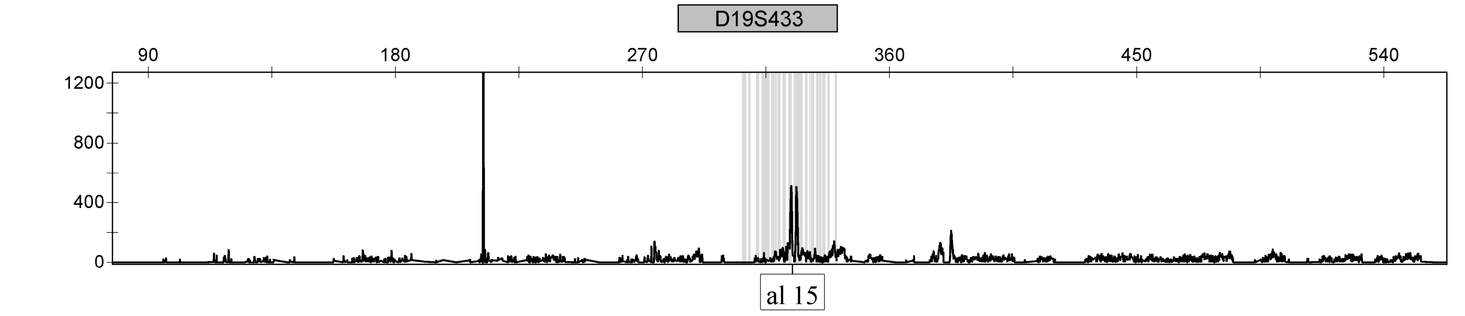

Applied Biosystems
GeneMapper 4.0

CellLineAuthentication-0326

Sample File	Sample Name	Panel	SQI	OS	SQ
57_A08_CellLineAuthentication-1-0402.FSA	6	STR Profile 4-human			

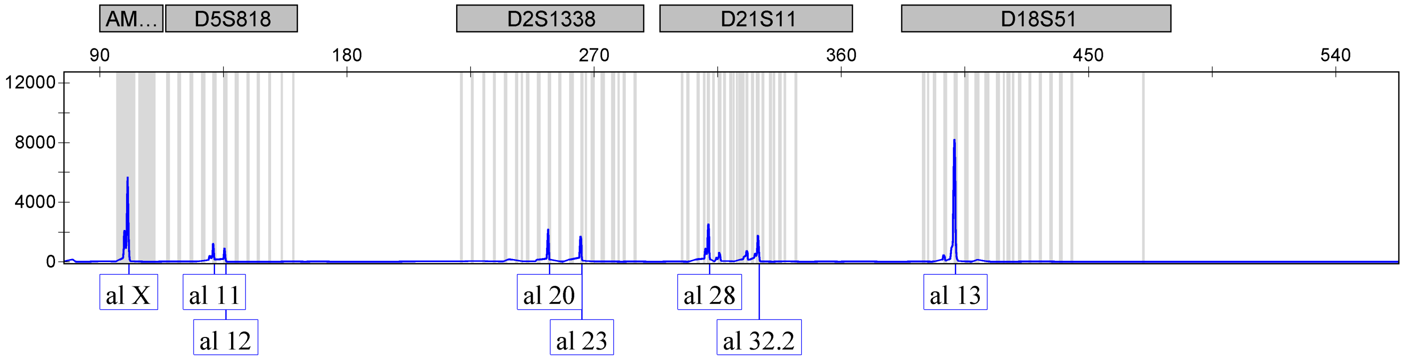

Sample File	Sample Name	Panel	SQI	OS	SQ
57_A08_CellLineAuthentication-1-0402.FSA	6	STR Profile 4-human			

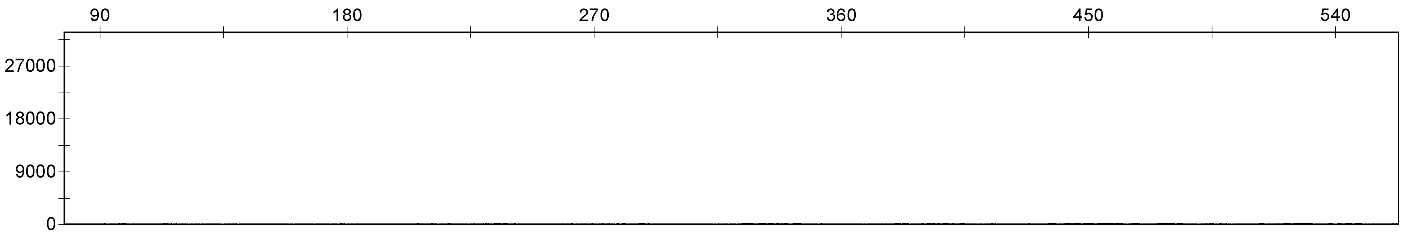

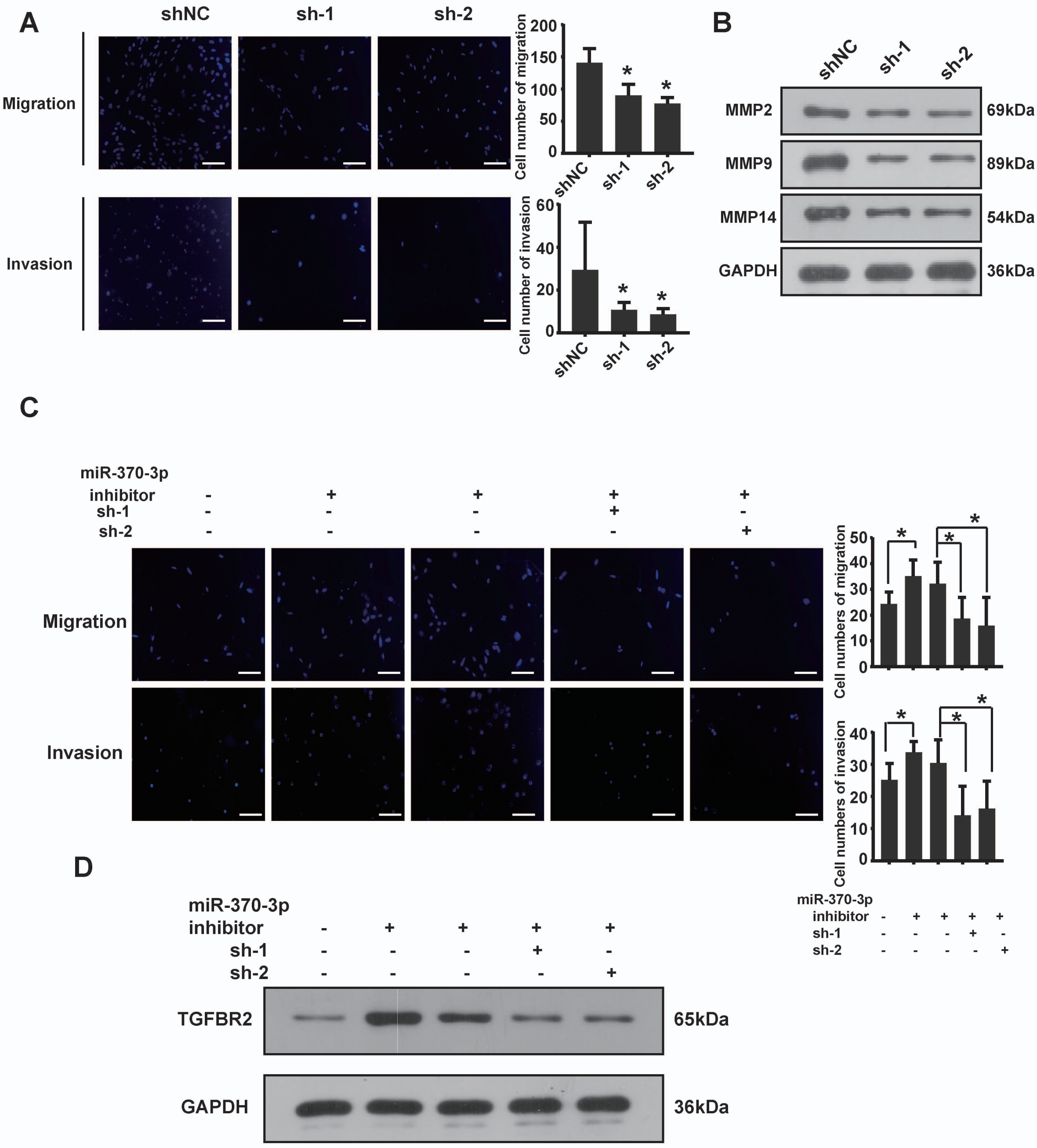
A
shNC
sh-1
sh-2
Migration
Invasion
Cell number of migration
Cell number of invasion
B
MMP2
MMP9
MMP14
GAPDH
69kDa
89kDa
54kDa
36kDa
C
miR-370-3p inhibitor
sh-1
sh-2
Cell numbers of migration
Cell numbers of invasion
D
TGFBR2
GAPDH
65kDa
36kDa

Supplementary Figure 1. **Decrease in circARID1A inhibited U118 cell migration and invasion via modulating miR-370-3p/TGFBR2 pathways. A**. Left: Images of Transwell assays of U118 cells, scale bar, 100μm; Right: Analyses of Transwell assay results indicated that decrease in circARID1A significantly inhibited U87 cell migration and invasion. n = 5,*p < 0.05, ANOVA. **B.** Images of western blotting showed that the expressions of MMPs decreased after silencing circARID1 **C**. Left: Images of Transwell assays of U118cells; Right: Analyses of Transwell assays results indicated that transfections of miR-370-3p inhibitor promoted U118 cell migration and invasion, while silencing circARID1A suppressed the promotion. scale bar, 50μm n = 5,*p < 0.05, ANOVA . **D**. Images of western blotting showed that expression of TGFBR2 protein was decreased after silencing circARID1A.
